# Corticosteroid-Induced Regression of Glioblastoma: A Radiographic Conundrum

**DOI:** 10.3389/fonc.2019.01288

**Published:** 2019-11-22

**Authors:** Joshua A. Cuoco, Brendan J. Klein, Christopher M. Busch, Evin L. Guilliams, Adeolu L. Olasunkanmi, John J. Entwistle

**Affiliations:** ^1^Section of Neurosurgery, Carilion Clinic, Roanoke, VA, United States; ^2^Virginia Tech Carilion School of Medicine, Roanoke, VA, United States; ^3^Virginia Tech School of Neuroscience, Blacksburg, VA, United States; ^4^Edward via College of Osteopathic Medicine, Blacksburg, VA, United States

**Keywords:** glioblastoma, astrocytoma, brain tumor, corticosteroid, dexamethasone, regression, vanishing tumor, neuro-oncology

## Abstract

Corticosteroid-induced regression of lesion contrast enhancement on imaging studies is most commonly appreciated with primary central nervous system lymphoma; however, although exceedingly rare, a limited number of primary and metastatic intracranial lesions have been reported to exhibit similar radiographic changes subsequent to corticosteroid therapy. To date, there have been six cases of glioblastoma reported to exhibit such changes. Lesion transformation on repeat imaging after the initiation of steroids represents a diagnostic dilemma for clinicians when attempting to differentiate between a diagnosis of glioblastoma and lymphoma. Stereotactic biopsy may be inadvertently postponed due to high clinical suspicion for steroid-induced cytotoxicity traditionally seen with lymphomatous cells. To highlight this radiographic conundrum, we present a rare case of corticosteroid-induced regression of glioblastoma and discuss the relevant literature. To our knowledge, this is the first case report to describe the molecular profile of a glioblastoma that underwent corticosteroid-induced regression.

## Background

Glioblastoma is the most common malignant brain tumor in adults with an estimated incidence of 2–3 cases per 100,000 persons worldwide (1). Despite surgical resection followed by adjuvant radiotherapy and chemotherapy, long-term survival from glioblastoma remains dismal with a median survival time of 1 year (1). Radiographically, glioblastoma typically presents as a solitary infiltrative supratentorial lesion with intense peripheral contrast enhancement, a variable degree of central necrosis, and surrounding vasogenic edema. On the contrary, primary central nervous system lymphoma (PCNSL) in an immunocompetent host appears as a supratentorial periventricular lesion with vivid homogeneous enhancement without central necrosis and a variable degree of vasogenic edema. Nevertheless, these lesions can often appear similar on routine morphological magnetic resonance imaging (MRI) emphasizing the necessity for definitive tissue diagnosis to establish appropriate treatment options.

Patients who harbor intracranial lesions are typically given a course of corticosteroids to reduce peritumoral edema and relieve associated neurologic symptomatology. Contrary to PCNSL, glioblastoma is not known to routinely undergo radiographic transformation after the initiation of systemic corticosteroid treatment. However, corticosteroid-induced regression of glioblastoma is a rare but documented phenomenon with six cases reported since first described in 1997 (2–6). Radiographic changes in glioblastoma following corticosteroid therapy demonstrate a diagnostic conundrum for clinicians as biopsy may be delayed due to clinical suspicion for PCNSL. Here, we present an atypical case of a 76-year-old immunocompetent male found to have a heterogeneous peripherally enhancing lesion of the right parietal lobe on imaging that underwent corticosteroid-induced regression on repeat pre-operative imaging 4 weeks later. Stereotactic biopsy revealed a histologic diagnosis of glioblastoma. To our knowledge, this is the first case report to describe the molecular profile of a glioblastoma that underwent corticosteroid-induced regression.

## Case Presentation

**Figure 1:**
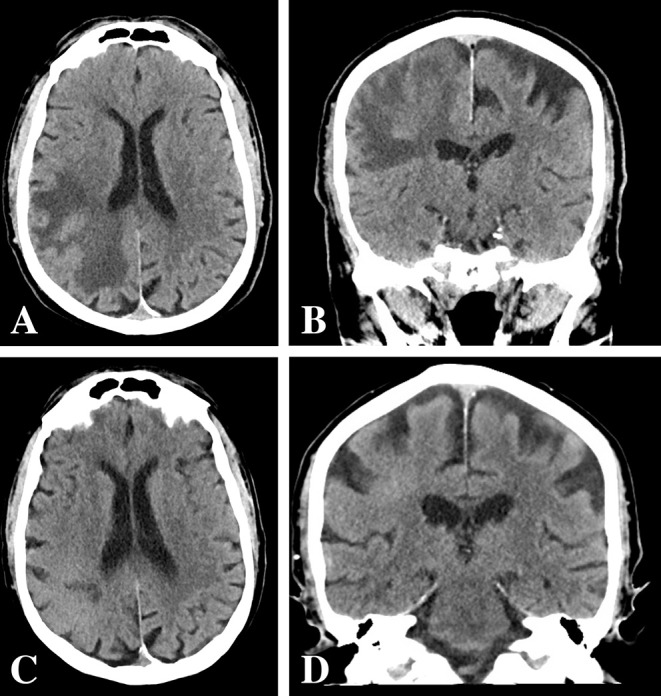
CT head pre- and post-corticosteroid therapy. **(A,B)** Initial CT head pre-corticosteroid therapy revealed a large hypodense lesion in the right parietal lobe with vasogenic edema. **(C,D)** Repeat CT head 4 weeks after post-corticosteroid therapy revealed a decrease in vasogenic edema and apparent size of the lesion.

**Figure 2:**
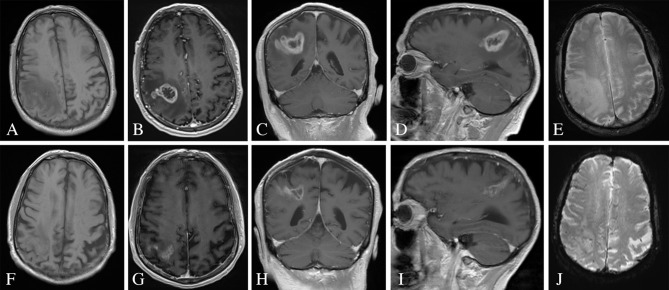
MRI of the brain pre- and post-corticosteroid therapy. **(A)** Initial T1-weighted imaging without evidence of subacute hemorrhage. **(B–D)** Initial MRI MP-RAGE sequences of the brain pre-corticosteroid therapy demonstrated a thick-walled peripherally enhancing lesion of the right parietal lobe with central necrosis and extensive surrounding vasogenic edema. **(E)** Initial gradient echo sequence without evidence of subacute hemorrhage. **(F)** Repeat T1-weighted imaging without evidence of subacute hemorrhage. **(G–I)** Repeat MRI MP-RAGE sequences of the brain 5 weeks after post-corticosteroid therapy demonstrated reduction in contrast enhancement, vasogenic edema, and apparent size of the lesion. **(J)** Repeat gradient echo sequence without evidence of subacute hemorrhage.

A 76-year-old immunocompetent Caucasian male presented to the emergency department with 1 week of left hand clumsiness. Physical examination was consistent with mild left upper extremity neglect but otherwise benign. The patient had no relevant past medical history. Computed tomography (CT) scan demonstrated a large hypodense lesion in the right parietal lobe suspicious for neoplasm (Figures 1A,B). Subsequently, MRI revealed a heterogeneous thick-walled peripherally enhancing lesion of the right parietal lobe with central necrosis and an extensive degree of surrounding vasogenic edema (Figures 2A–E). Neither T1-weighted imaging nor gradient echo sequences demonstrated evidence of subacute hemorrhage. Initial differential diagnosis included glioblastoma, anaplastic astrocytoma, metastasis, or PCNSL. A metastatic workup was negative. The patient was started on dexamethasone (16 mg per day for 5 days) while hospitalized with improvement in his presenting neurologic symptomatology. Upon discharge, he was prescribed a 6-day dexamethasone taper to 1 mg per day to continue until outpatient follow up. Per patient preference, stereotactic debulking of the lesion was scheduled for 5 weeks from his initial presentation.

**Figure 3:**
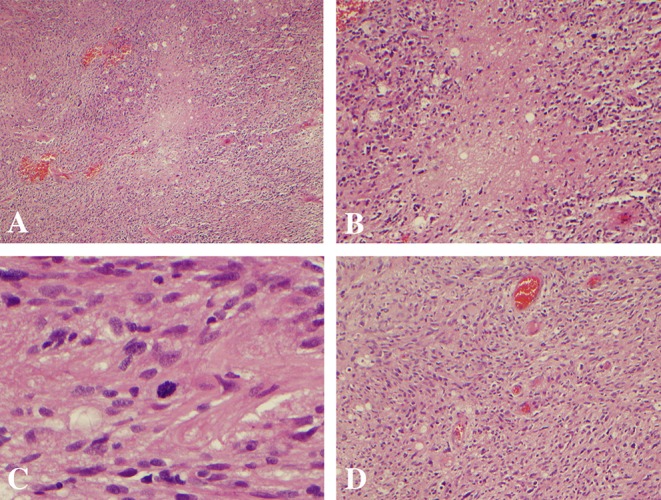
Histopathology demonstrating pleomorphic glial cells, nuclear atypia, palisading necrosis, mitoses, and vascular proliferation consistent with a diagnosis of glioblastoma. **(A,B)** Palisading necrosis at 4x and 10x magnification, respectively. **(C)** Mitoses at 40x magnification. **(D)** Vascular proliferation at 10x magnification.

The patient was admitted to the hospital 4 weeks later (1 week prior to scheduled surgery) with new onset lethargy and respiratory distress. Repeat CT compared to 4 weeks prior demonstrated a dramatic reduction in vasogenic edema and apparent size of the lesion (Figures 1C,D). The patient was diagnosed with bacterial pneumonia and remained hospitalized for medical treatment and optimization. One week later, a repeat MRI was ordered to evaluate the significant changes seen on repeat CT as well as to determine extent of future operative intervention. Repeat MRI compared to 5 weeks prior revealed a dramatic reduction in contrast enhancement, vasogenic edema, and apparent size of the lesion (Figures 2F–J). These imaging findings raised suspicion for PCNSL. As such, a stereotactic biopsy was planned to attain tissue diagnosis. Biopsy samples were sent as frozen sections to surgical pathology, which revealed pleomorphic glial cells, nuclear atypia, microvascular proliferation, and palisading necrosis consistent with a diagnosis of glioblastoma (Figure 3). The decision was then made to proceed with debulking of the tumor.

**Figure 4:**
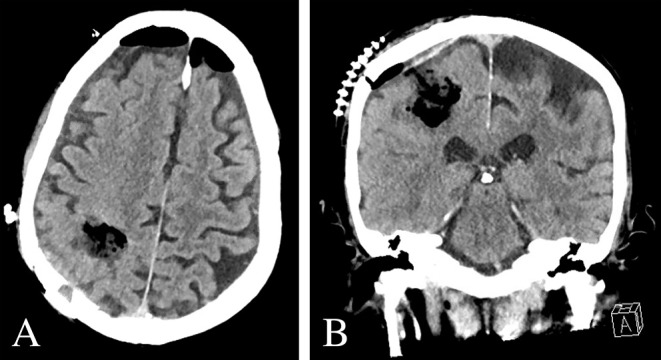
Post-operative imaging. **(A,B)** Post-operative CT Head demonstrated expected post-operative changes without evidence of acute pathology.

Molecular genetic studies revealed wildtype isocitrate dehydrogenase (IDH) 1 and 2, promoter methylation of O^6^-methylguanine DNA methyltransferase (MGMT), non-amplified epidermal growth factor receptor (EGFR) by fluorescence *in situ* hybridization, and poor p53 expression with 1% tumor cell staining on immunohistochemistry. Adjuvant radiotherapy and chemotherapy were planned to start 3 weeks post-operatively. However, the patient developed worsening lethargy and fatigue 2 weeks after his operation. Post-operative imaging was unrevealing for acute intracranial pathology (Figure 4). Infectious workup remained negative; however, he failed to improve clinically. As such, his clinical demise was attributed to rapid disease progression of recently diagnosed glioblastoma. He was transferred to hospice care and expired 1 month from the date of initial surgery.

## Discussion

Corticosteroid-induced regression on imaging is a feature most commonly associated with PCNSL; however, this characteristic has also been reported in cases of metastatic renal cell carcinoma, medulloblastoma, and glioblastoma (2–8). It is thought that corticosteroids cause lymphomatous neoplasms of the brain to undergo rapid cell cycle arrest and cell death, most notably via a p38 mitogen-activated protein kinase-dependent mechanism in T and B cells (9–11). Contrary to PCNSL, corticosteroids do not exhibit tumoricidal effects against glioblastoma nor do they considerably alter their size; rather, dexamethasone decreases vasogenic edema associated with glioblastoma via reducing the permeability of capillaries within the blood brain barrier (12). Corticosteroids can significantly reduce neurologic symptomatology associated with glioblastoma as some studies have shown a 1-week course of corticosteroids to reduce peritumoral edema by as much as 50% (13). Although corticosteroids reduce apparent tumor volume, MRI volumetric studies have demonstrated this reduction to be restricted to vasogenic edema with tumor size remaining relatively constant (14).

Corticosteroid-induced regression of glioblastoma is an exceedingly rare phenomenon. Indeed, our literature search revealed only six cases of this entity since first reported in 1997 (Table 1) (2–6). Buxton et al. first described a case of a disappearing frontoparietal glioblastoma in a 56-year-old male after a short course of dexamethasone (6 mg per day, unspecified duration) (2). Pre-operative imaging 3 weeks thereafter demonstrated disappearance of the lesion. The patient was taken off dexamethasone and biopsy was rescheduled for 3 weeks later. Repeat imaging demonstrated reappearance of the frontoparietal lesion consistent with radiographic characteristics seen in PCNSL. However, biopsy confirmed a histopathologic diagnosis of glioblastoma. In 2004, Zaki et al. described two similar cases of multicentric glioblastoma, both of which demonstrated lesions in the right parietal lobe and splenium of the corpus callosum (3). Repeat imaging after a course of dexamethasone (16 mg per day for 3 weeks) revealed reduced contrast enhancement in the parietal lesions with increased enhancement within the splenial lesions in both cases. These patients were taken off dexamethasone and repeat imaging was ordered for 3 weeks later. One case demonstrated reappearance of both lesions whereas the second case demonstrated reappearance of only the splenial lesion. Similarly, Goh et al. reported a case of multicentric glioblastoma with lesions in the right temporal lobe and splenium; however, after cessation of dexamethasone, a new focus of contrast enhancement within the right frontal lobe was demonstrated in addition to reappearance of the original lesions (5). Moreover, Mazur et al. described reappearance of a temporoparietal lesion with new onset leptomeningeal carcinomatosis after cessation of dexamethasone (6). In their report, the authors described the reduction in lesion enhancement followed by lesion reappearance after stopping dexamethasone as corticosteroid-induced “pseudoregression.”

**Table 1:** Summary of reported cases of corticosteroid-induced regression of glioblastoma.

**Years**	**Author**	**Age/Sex**	**Multicentric**	**Original location(s)**	**Radiographic change**	**Time to reappearance**	**Location of reappearance**	**Dexamethasone dose**	**Treatment**	**Clinical** **outcome**	**Molecular** **profile**	**References**
1997	Buxton et al.	56 M	No	Left frontoparietal	Disappearance of lesion and enhancement	3 weeks	Same	6 mg per day, unspecified duration	None	Death immediately subsequent to biopsy	NR	(2)
2004	Zaki et al.	53 M	Yes	Right parietal, splenium	Reduced enhancement in parietal lesion, increased splenial enhancement	3 weeks	Same	16 mg per day for 3 weeks	Radiotherapy	NR	NR	(3)
2004	Zaki et al.	75 M	Yes	Right parietal, splenium	Reduced enhancement in parietal lesion, increased splenial enhancement	3 weeks	Splenium only	16 mg per day for 3 weeks	None	Death before radiation, unspecified timing	NR	(3)
2009	Hasegawa et al.	59 M	No	Left parietal	Reduced enhancement	4 weeks	Same	16 mg per day for 4 weeks	NR	NR	NR	(4)
2009	Goh et al.	61 F	Yes	Right temporal, splenium	Near resolution of all lesions	4 weeks	Same plus new right frontal lesion	16 mg per day for 4 weeks	Radiotherapy	Death ~4 months after reappearance	NR	(5)
2012	Mazur et al.	57 F	No	Right temporoparietal extending into splenium	Reduced enhancement	2 weeks	Same plus leptomeningeal carcinomatosis	16 mg per day for 5 days	Radiotherapy, temozolomide	Alive 2 months after radiographic change	NR	(6)
2019	Cuoco et al.	76 M	No	Right parietal	Reduced enhancement	N/A	N/A	16 mg per day for 5 weeks tapered	None	Death 1 month after surgery	Wildtype IDH1/2 Methylated MGMT Non-amplified EGFR Poor p53 expression	

*M, male; NR, not reported; F, female; N/A, not applicable; IDH, isocitrate dehydrogenase; MGMT, O^6^-methylguanine DNA methyltransferase; EGFR, epidermal growth factor receptor*.

Collectively, the mean age at time of diagnosis in these six cases was 60.2 years (range: 53–75 years) with a slight male predilection (4 of 6 cases). Half of the cases reported multicentric glioblastoma. The parietal lobe was the most common original location of glioblastoma involved in 5 of 6 cases. The average duration of dexamethasone therapy was 2.9 weeks (range 5 days−4 weeks) with the majority of cases prescribing 16 mg per day. Overall, clinical outcome documented in 4 of 6 cases was dismal with the longest documented survival time reported to be 4 months from lesion reappearance on imaging. With the most recent case published in 2012, none of these cases documented results of molecular genetic studies. The case we present has similar epidemiologic and clinical characteristics as those previously reported including age (76 years old vs. a mean of 60.2 years old), sex predilection (male), original location of the tumor (parietal lobe), and clinical outcome (death only months after surgery). Contrary to the cases described in prior, we chose to pursue stereotactic biopsy immediately after repeat imaging despite reduced lesion enhancement in order to attain tissue diagnosis and expedite treatment options (2–6). Molecular genetic studies from our case revealed wildtype IDH1/2, methylated MGMT, non-amplified EGFR, and poor p53 expression. These data establish the first molecular profile of a glioblastoma that underwent corticosteroid-induced regression.

With the exception of PCNSL, the only other primary intracranial lesion, to our knowledge, that has been reported to exhibit corticosteroid-induced regression is a case of a medulloblastoma described by Gupta et al. (8). The authors described a 17-year-old female with a poorly defined contrast-enhancing lesion within the posterior fossa with associated hydrocephalus and effacement of the fourth ventricle (8). The patient was started on dexamethasone (12 mg per day for 2 weeks) and underwent a single fraction of radiotherapy (8 Gy). Three weeks later, a suboccipital craniectomy was performed for planned tumor resection; however, despite multiple biopsies, no tumor or inflammatory lesion was identified. Post-operative CT was without evidence of the lesion. Follow up imaging 5 months later revealed a heterogeneously enhancing lesion within the left cerebellar hemisphere with cystic and solid components. Biopsy was repeated and histopathology confirmed a diagnosis of medulloblastoma. Similar to the majority of intracranial lesions, medulloblastoma is not subject to steroid-induced toxicity nor are these lesions known to exhibit radiographic changes on subsequent imaging after the initiation of steroids. This case illustrates that corticosteroids should be stopped and repeat imaging should be obtained if a lesion cannot be localized macroscopically via open craniotomy or diagnosed microscopically via frozen pathology.

## Conclusions

As depicted by the present case and prior reports, the degree of glioblastoma regression with dexamethasone can be substantial and confound diagnostic clinical suspicion. As such, this case not only illustrates the importance in obtaining a contrast-enhanced CT or MRI scan in a reasonably close timeframe to performing neurosurgical intervention but also emphasizes the necessity in acquiring tissue diagnosis for intracranial lesions that transform in appearance on imaging studies. Indeed, pathologic diagnosis is crucial as corticosteroid-induced changes can represent steroid-induced tumor cytotoxicity in PCNSL or a rare unknown transformative process in glioblastoma. In order to better understand their natural history, the molecular profile of glioblastoma that regress with corticosteroid therapy must be established.

## Ethics Statement

Written informed consent was obtained from the individual(s) for the publication of any potentially identifiable images or data included in this article.

## Author Contributions

JC: primary author of manuscript. JC, BK, CB, EG, AO, and JE: provided substantial contributions to the conception and design of the manuscript, contributed to manuscript revision, read and approved the submitted version, and agree to be accountable for all aspects of the work ensuring that questions related to the accuracy or integrity of any part of the work are investigated and resolved.

### Conflict of Interest

The authors declare that the research was conducted in the absence of any commercial or financial relationships that could be construed as a potential conflict of interest. The handling editor declared a past supervisory role with one of the author JC.

## Abbreviations

PCNSL: primary central nervous system lymphoma
MRI: magnetic resonance imaging
CT: computed tomography
IDH: isocitrate dehydrogenase
MGMT: O^6^-methylguanine DNA methyltransferase
EGFR: epidermal growth factor receptor.
